# Gut Microbiota-Regulated Pharmacokinetics of Berberine and Active Metabolites in Beagle Dogs After Oral Administration

**DOI:** 10.3389/fphar.2018.00214

**Published:** 2018-03-21

**Authors:** Ru Feng, Zhen-Xiong Zhao, Shu-Rong Ma, Fang Guo, Yan Wang, Jian-Dong Jiang

**Affiliations:** State Key Laboratory of Bioactive Substance and Function of Natural Medicines, Institute of Materia Medica, Chinese Academy of Medical Sciences and Peking Union Medical College, Beijing, China

**Keywords:** berberine, gut microbiota, butyrate, metabolites, GC-MS, LC/MS^n^-IT-TOF, 16S rRNA genes analysis

## Abstract

Berberine (BBR) is considered a multi-target drug that has significant advantages. In contrast to its significant pharmacological effects in clinic, the plasma level of BBR is very low. Our previous work revealed that dihydroberberine (dhBBR) could be an absorbable form of BBR in the intestine, and butyrate is an active metabolite that is generated by gut bacteria in rats. In this study, for the first time we describe gut microbiota-regulated pharmacokinetics in beagle dogs after oral administration of BBR by single (50 mg/kg) or multiple doses (50 mg/kg/d) for 7 days. GC-MS, GC, LC-MS/MS, and LC/MS^n^-IT-TOF were used to detect dhBBR, butyrate and BBR as well as its Phase I and II metabolites, respectively. The results showed that dhBBR was not detected in dog plasma but was excreted in small amounts in the feces of dogs examined on days 3 and 7. Butyrate was generated by gut bacteria and increased by 1.3- and 1.2-fold in plasma or feces, respectively, after 7 days of BBR treatment compared to the levels before treatment. Changes of intestinal bacterial composition were analyzed by 16S rRNA genes analysis. The results presented that dogs treated with BBR for 7 days increased both the abundance of the butyrate- and the nitroreductases- producing bacteria. We also identified chemical structures of the Phase I and II metabolites and analyzed their contents in beagle dogs. Eleven metabolites were detected in plasma and feces after BBR oral administration (50 mg/kg) to dogs, including 8 metabolites of Phase I and III metabolites of Phase II. The pharmacokinetic profile indicated that the concentration of BBR in plasma was low, with a *C*_*max*_ value of 36.88 ± 23.45 ng/mL. The relative content of glucuronic acid conjugates (M11) was higher than those of other metabolites (M1, M2, M12, and M14) in plasma. BBR was detected in feces, with high excreted amounts on day 3 (2625.04 ± 1726.94 μg/g) and day 7 (2793.43 ± 488.10 μg/g). In summary, this is the first study to describe gut microbiota-regulated pharmacokinetics in beagle dogs after oral administration of BBR, which is beneficial for discovery of drugs with poor absorption but good therapeutic efficacy.

## Introduction

**Figure 1:**
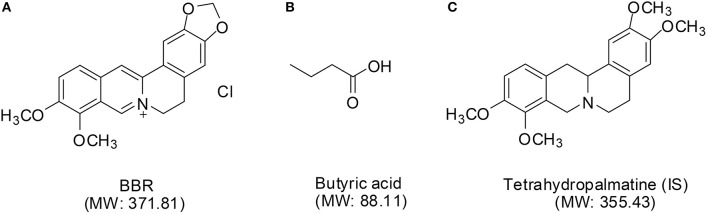
The chemical structures of BBR **(A)**, butyric acid **(B)**, and IS **(C)**.

Berberine (BBR) (its structure is shown in Figure 1A) is an isoquinoline alkaloid derived from the rhizome of *Coptis chinensis* (“Huang-Lian” in Chinese) of the Ranunculaceae family. It has been widely used throughout history as an antidiarrheic. In recent years, other bioactivities have been discovered for BBR, including antidiabetic (Yin et al., 2012), anticancer (Iizuka et al., 2000), cardioprotective (Wang et al., 1994), anti-inflammatory (Chang et al., 1990), and especially, anti-hyperlipidemia (Zhang et al., 2008, 2010; Derosa et al., 2013; Dong et al., 2013) effects, that lower the total cholesterol (TC), triglyceride (TG), and low-density-lipoprotein cholesterol (LDL-*c*) levels in patients (Kong et al., 2004; Li et al., 2011).

Recently, the gut microbiota has been considered to be a “hidden organ” of the body and may be associated with the pathogenesis of diseases, such as cardiovascular diseases, obesity, and diabetes (Wang et al., 2011; Tremaroli and Bäckhed, 2012; Koeth et al., 2013). Butyrate (structure shown in Figure 1B), as one of the most extensively examined short-chain fatty acids (SCFA) (Byrne et al., 2015; Koh et al., 2016), is the principal product of bacterial fermentation in the gut and may regulate host energy metabolism and benefit health (Gao et al., 2009; Natarajan and Pluznick, 2014). Studies have shown that BBR modulates the composition of the intestinal bacterial community (Zhang et al., 2012), and our previous study demonstrated that butyrate is generated by oral administration of BBR and improves energy metabolism in the presence of berberine (Wang et al., 2017a).

However, in contrast with the significant pharmacological effects of BBR in the clinic, the plasma level of BBR is very low (Hua et al., 2007), and its absolute bioavailability has been reported to be less than 1% (Chae et al., 2008; Liu et al., 2010; Chen et al., 2011). To explain this discrepancy and obtain more information about its metabolic characteristics *in vivo*, in this study, we studied the gut microbiota-regulated pharmacokinetics in beagle dogs after oral administration of berberine. GC-MS, GC, LC-MS/MS, and LC/MS^n^-IT-TOF were used to determine BBR and its active metabolites, especially those generated by the gut microbiota. We also performed the bacterial composition analysis by 16S rRNA genes analysis and QIIME. The results of this study will benefit drug discovery by offering a pharmacokinetic profile of drugs with poor absorption but good therapeutic efficacy.

## Materials and methods

### Chemicals and reagents

BBR was obtained from J&K Scientific Ltd. (Beijing, China). Tetrahydropalmatine, as an internal standard (IS, structure shown in Figure 1C), was purchased from the National Institute for the Control of Pharmaceutical and Biological Products (Beijing, China). Dihydroberberine (dhBBR), thalifendine (M1), berberrubine (M2), demethylenberberine (M3), jatrorrhizine (M4), and palmatine (M5) were supplied from Chengdu Must Bio-technology Co., Ltd. (Chengdu, China). The purity of all of the above standards was more than 98%. Butyric acid (99.9%) was obtained from the Sigma-Aldrich Corporation (Saint Louis, USA). Phosphoric acid was analytical grade and was purchased from Sinopharm Chemical Reagent Co., Ltd. (Beijing, China). Chromatographic grade acetone was purchased from Sinopharm Chemical Reagent Co., Ltd. (Beijing, China). HPLC- or LC-MS-grade acetonitrile was obtained from Fisher Scientific Ltd. (Fair Lawn, NJ). Deionized distilled water was Wahaha purified (Hangzhou Wahaha Group Co., Ltd., China). All of the other chemical reagents were of the highest grades available from Sinopharm Chemical Reagent Co., Ltd. (Beijing, China).

### Animals

Five male beagle dogs (8–10 kg) were supplied by Beijing Marshall Biotechnology Co., Ltd. (Beijing, China). Animals were housed in cage racks and had free access to food and water with a 12 h light/dark cycle (light on from 8:00 a.m. to 8:00 p.m.) at ambient temperature (22–24°C) and 45% relative humidity. Dogs were fasted for 12 h before all experimental studies. All experiments were conducted in accordance with institutional and ethics guidelines and were approved by the Laboratories Institutional Animal Care and Use Committee of the Chinese Academy of Medical Sciences & Peking Union Medical College.

### Instruments

Gas chromatograph-mass spectrometry (GCMS-QP2020, Shimadzu Cooperation, Japan) and liquid chromatography-triple quadrupole mass spectrometry (LC-MS/MS 8050, Shimadzu Cooperation, Japan) were used to determine and quantify dhBBR and BBR and the Phase I and II metabolites in plasma and feces of beagle dogs. A gas chromatograph (GC-2014, Shimadzu Cooperation, Japan) equipped with a flame ionization detector (FID) was used to analyze butyrate. High-performance liquid chromatography coupled to ion trap time-of-flight mass spectrometry (LCMS-IT-TOF, Shimadzu Cooperation, Japan) was applied to identify BBR and its metabolites.

Instruments for bacterial Composition Analysis were including: Gene JET Gel Extraction Kit (QIAGEN, Germany), NEXTflex Rapid Illumina DNA-seq Kit (Ipswich, MA), Qubit 2.0 Fluorometer (Thermo Scientific, Carlsbad, CA), Agilent Bioanalyzer 2100 system (Agilent Technologies) and HiSeq2500 (Illumina).

### Determination of dhBBR in plasma and feces by GC-MS

#### Experimental conditions

The GCMS-QP2020 was used to analyze BBR metabolites and dhBBR. An Alltech (GRACE ALLTECH, America) capillary column (AT TM−1701, 30 m × 0.25 mm × 0.25 μm) was operated in the splitless mode. The helium carrier flow was 39.7 cm/s under a column head pressure of 68.1 kPa. The oven temperature was initially 50°C for 2 min and was gradually increased to 260°C at a rate of 8°C/min and maintained for 25 min. The injector and detector temperatures were 280 and 230°C, respectively. The mass spectra were recorded over a scan range of 60–800 m/z. Structural identification of possible metabolites was based on matching with standard mass spectra available in the Shimadzu GC-MS library, and m/z 337.0 was selected for quantification of dhBBR.

#### Preparation of plasma and feces sample

For the determination of dhBBR obtained from plasma and feces, a working solution of dhBBR was prepared at concentrations of 500, 200, 100, 50, 25, 10, 5, and 1 μg/mL by diluting the stock solution (1 mg/mL) with methanol, and these concentrations were used to generate the standard curve. The initial dhBBR stock solution (1 mg/mL) was prepared in DMSO. Plasma samples 100 μL were prepared by adding 1 mL of ethyl acetate and mixing twice. Then, the superstrata were collected and evaporated to dryness using a rotary evaporator. The residues were dissolved in methanol (100 μL) and vortex-mixed for 3 min, followed by centrifugation (at 14,000 g for 5 min). The superstrata were injected into the GC-MS. Feces samples were mixed with 4-fold (g/mL) distilled water-acetonitrile (1:1) and prepared by adding 8-fold ethyl acetate, while the other procedures were the same as the plasma processing method.

### Determination of butyrate in plasma and feces by GC

#### Experimental conditions

The sample was injected at a volume of 1 μL in splitless mode onto a high polarity Alltech capillary column (AT-WAX, 30 m × 0.25 mm × 0.25 μm, Alltech company, ME). Nitrogen was used as the carrier gas at a flow rate of 1.27 mL/min, with a purge flow setting of 3.0 mL/min. The nitrogen carrier flow was 56.0 cm/s under a column total pressure of 105.0 kPa. The initial oven temperature was 80°C, which was maintained for 1 min, and then gradually increased to 130 at a rate of 5°C/min, which was maintained for 5 min. The injection port and FID detector temperatures were 230 and 250°C, respectively. The running time for each analysis was 18 min.

#### Preparation of plasma and feces samples

Each plasma sample (100 μL) was mixed with an equal volume of acetone (1% phosphoric acid, v/v) for 3 min. The mixture was centrifuged at 10,000 g/min for 5 min at 4°C, and then, the supernatant was directly injected into the GC system for analysis.

Each feces sample was combined with 8-fold (g/mL) acetone (1% phosphoric acid, v/v), vortexed until the mixture was uniform, and then kept at room temperature for 2 h. The supernatant was collected as feces homogenates. Next, the mixture was centrifuged at 10,000 g/min for 5 min at 4°C, and the supernatant was then directly injected into the GC system for analysis.

#### Validation of the GC method for determination of butyrate

##### Calibration standards and QC preparation

Calibration standards were prepared at series concentrations of 0.625, 1.25, 2.5, 5, 12.5, 25, 50, 100, 200, and 400 μg/mL by diluting the stock solution with acetone (1% phosphoric acid, v/v). The three levels used to validate the accuracy and precision were 1.25, 50, and 400 μg/mL (LQC, MQC and HQC).

##### Method validation

The method validation assays were carried out according to the currently accepted Chinese State Food and Drug Administration (SFDA) bioanalytical method validation guidelines. Selectivity, linearity, precision, accuracy, recovery and stability were assayed.

### Qualitative and quantitative determinations of BBR metabolites in plasma and feces by LC-MS/MS and LC/MS^n^-IT-TOF

#### Experimental conditions

LC-MS/MS with an Alltima C_18_ column (Shim-pack XR-ODS II 75 L × 2.0, Shimadzu, Japan) was employed to analyze BBR and its metabolites in biological samples. Samples were eluted through the column with a gradient of water-formic acid (100:0.5, v/v) and acetonitrile (0.00 min, 90:10; 2.00 min, 80:20; 4.00 min, 75:25; 5.00 min, 70:30; 5.01 min, 20:80; 6.00 min, 10:90; 6.01 min, 90:10; 8.00 min, 90:10) at a flow rate of 0.4 mL/min at 30°C. For MS analysis, an ESI resource in positive mode was used, and the other parameters were as follows: the nebulizing gas flow was 3.0 L/min; heating gas flow and drying gas flow were 10.0 L/min; interface temperature was 250°C; DL temperature was 300°C; and heat block temperature was 400°C. For full-scan MS analysis, the spectra were recorded in the range of m/z 100–1,000.

The MS/MS transitions and capillary voltage (CE) conditions were as follows: m/z 335.70→ m/z 320.10 for BBR; m/z 355.70→ m/z 191.90 for IS; m/z 321.65→ m/z 307.15 for M1; m/z 321.65→ m/z 307.15 for M2; m/z 324.10→ m/z 308.20 for M3; m/z 337.70→ m/z 322.10 for M4; m/z 353.05→ m/z 337.20 for M5; m/z 338.00→ m/z 323.11 for M6; m/z 324.00→ m/z 309.00 for M7; m/z 352.00→ m/z 337.00 for M8; m/z 310.00→ m/z 295.00 for M9/M10; m/z 498.00→ m/z 322.00 for M11/M12; m/z 402.00→ m/z 322.00 for M13; m/z 514.00→ m/z 338.00 for M14; m/z 500.00→ m/z 324.00 for M15; m/z 676.00→ m/z 500.00 for M16.

LC/MS^n^-IT-TOF with an Alltima C_18_ column (150 mm × 4.6 mm, 5 μm *i.d*., Alltech Cooperation, USA) was employed to analyze BBR and its metabolites in feces. Samples were eluted through the column with a gradient of water-formic acid (100:0.5, v/v) and acetonitrile (0 min, 90:10; 10 min, 75:25; 15 min, 60:40; 18 min, 50:50; 21.0 min, 30:70; 24 min, 5:95; 24.1 min, 90:10; 30 min, 90:10) at a flow rate of 0.8 mL/min at 40°C. For IT-TOF analysis, an ESI resource with positive mode was used, and other parameters were listed as follows: CDL temperature, 200°C; Heat block temperature, 200°C; Detector voltage, 1.56 kV; Nebulizing gas, 1.5 L/min; Collision energy, 50%; Drying gas pressure, 120 kPa. For full-scan MS analysis, the spectra were recorded in the range of m/z 100-900. Shimadzu LCMS solutions (3.60.361) were used for data acquisition and processing. The structures of BBR and its metabolites were characterized by the information provided by LC/MS^n^-IT-TOF.

#### Preparation of plasma and feces samples

Plasma (50 μL) was precipitated with 200 μL of methanol (with 10 ng/mL of IS). After vortexing, tubes were centrifuged at 14,800 rpm for 10 min. An aliquot of 10 μL was injected into the LC-MS/MS 8050 system.

Each feces sample was porphyrized by sonication for 30 min after adding methanol (1/10 g/mL). The supernatant was diluted to appropriate percentages for quantitative determination. Then, feces samples (50 μL) were precipitated with 200 μL of methanol (with 10 ng/mL of IS). After vortexing, tubes were centrifuged at 14,800 rpm for 10 min. An aliquot of 10 μL was injected into the LC-MS/MS 8050 system.

#### Method validation of LC-MS/MS for determination of BBR and its metabolites

##### Calibration standards and QC preparation

Plasma (50 μL) was mixed with 5 μL of working solutions of the calibration standards and then precipitated with 200 μL of methanol (with 10 ng/mL of IS). After vortexing, tubes were centrifuged at 14,800 rpm for 10 min. The standard curve concentrations were as follows: 0.1, 0.2, 0.4, 2, 10, 50, 100, and 200 ng/mL. QCs were prepared similarly. The concentrations of QC for M1-M5 were 0.2 ng/mL (low concentration of quality control, LQC), 10 ng/mL (median concentration of quality control, MQC), and 160 ng/mL (high concentration of quality control, HQC), and the concentrations of BBR were 0.4 (LQC), 10 (MQC), and 160 (HQC) ng/mL.

##### Method validation

The method validation assays were carried out according to the currently accepted Chinese State Food and Drug Administration (SFDA) bioanalytical method validation guidelines. Selectivity, linearity, precision, accuracy, extraction recovery, matrix effect and stability were assayed.

### Bacterial composition analysis

The 16S rRNA genes were amplified using the specific primer of 16S V3-V4: 340F-805R to target the V3-V4 regions of 16S rRNA. PCR products were mixed in equal ratios. Then, the mixture of PCR products was purified with a GeneJET Gel Extraction Kit. Sequencing libraries were generated by using a NEXTflex Rapid Illumina DNA-seq Kit from New England Biolabs following manufacturer's recommendations and adding index codes. The library quality was assessed on a Qubit 2.0 Fluorometer and Agilent Bioanalyzer 2100 system. Finally, the library was sequenced on a HiSeq2500 platform and 250 bp paired-end reads were generated. Sequences were analyzed using the Quantitative Insights Into Microbial Ecology (QIIME) software package. First, the QIIME quality filters categorized the reads. Then, we picked a representative sequence for each operational taxonomic unit (OTU) and used the ribosomal database project classifier to annotate taxonomic information for each representative sequence. Sequences with ≥97% similarity were assigned to the same OTUs.

### Collection of plasma and feces samples

Beagle dogs were orally administered identical dosage of BBR (50 mg/kg every day) for 7 days. Plasma and feces samples were collected at 3, 5, and 7 days. After at least 1 month of washout time, a single dose of BBR (50 mg/kg) was administrated to beagle dogs. Then, plasma samples (0.5 mL) were obtained from the foreleg venous plexus and transferred to heparinized tube at 5, 10, 20 in, 30 min, 1, 1.5, 2, 3, 4, 6, 8, 12, 24, 48 h. The test samples were prepared and analyzed as mentioned above. Dogs had free to access food and water 3 h after administration. Plasma and feces were stored at −20°C before analysis. All animal protocols were approved by the Animal Care and Welfare Committee of Institute of Materia Medica, Chinese Academy of Medical Sciences and Peking Union Medical College (Beijing, China). In addition, all animal experiments were conducted in strict accordance with the guidelines for the care and use of laboratory animals issued by the Institute Animal Care and Welfare Committee.

### Data analysis

All data were statistically analyzed using Prism 5.0 and are presented as the means ± standard deviation (SD). Das 3.0 was performed to calculate the plasma pharmacokinetic parameters.

The relative quantities of M11, M12, and M14 in plasma were correspondingly calculated by the standard curves of M1, M2, and M4. The relative quantities of M7, M9 and M10 in feces were calculated by a standard curve of M3, with M8 referred to the standard curve of M1.

## Results

### Determination of dhBBR in plasma and feces by GC-MS

**Figure 2:**
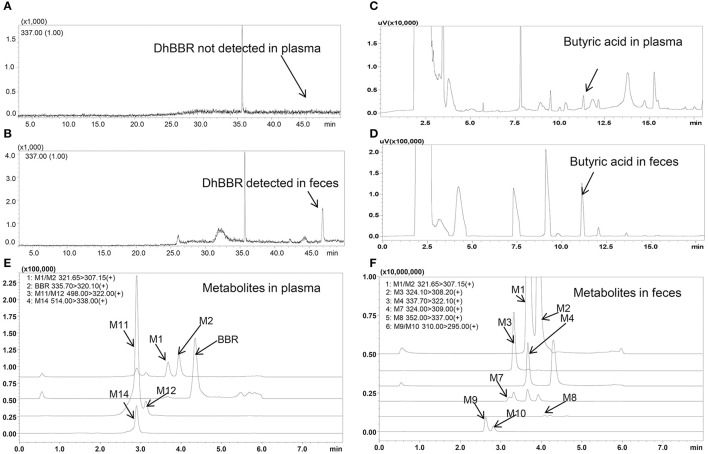
Spectra of butyric acid, dhBBR and its metabolites in beagle dog plasma and feces (**A**: DhBBR not detected in plasma; **B**: DhBBR detected in feces; **C**: Butyrate detected in plasma; **D**: Butyrate detected in feces; **E**: Metabolites detected in plasma; **F**: Metabolites detected in feces).

**Figure 3:**
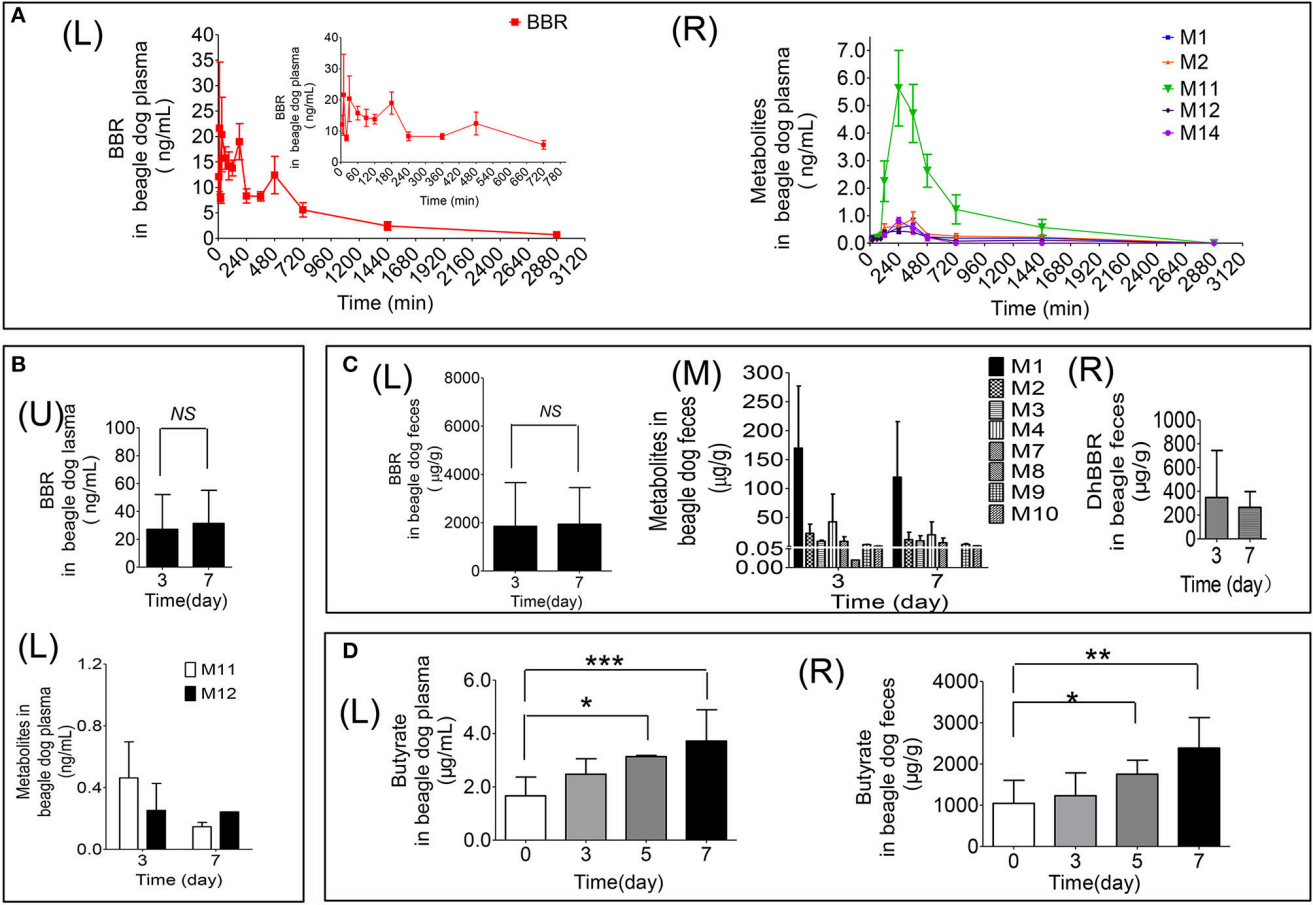
Quantitative data for BBR and its metabolites in beagle dogs (**A**: Contents of BBR **(L)** and its metabolites **(R)** in plasma (0–48 h); **B**: Contents of BBR **(U)** and its metabolites **(L)** in plasma (days 3 and 7); **C**: Contents of BBR **(L)**, its metabolites **(M)** and dhBBR **(R)** in feces (days 3 and 7); **D**: The quantitative graphs of butyrate in beagle dog plasma **(L)** and feces **(R)** after successive administration for 7 days, ^*^P < 0.05, ^**^P < 0.01 and ^***^P < 0.001, *n* = 5).

DhBBR was not detected in plasma by GC-MS after BBR oral administration to dogs (Figure 2A), but it was detected in feces (Figure 2B) at 348.0 ± 394.7 and 264.3 ± 133.0 μg/g on days 3 and 7, respectively (Figures 3C–R). This result was similar to that in SD rats reported in a previous work (Feng et al., 2015).

### Determination of butyrate in plasma and feces by GC

#### Validation of GC quantitation

The butyric acid spectra are shown in Figures 2C,D, and the chromatographic peak was sensitive and special without interference.

A linear relationship was found between the peak area and butyric acid concentrations over the ranges of 0.625–400 μg/mL. The linearity range was wide enough to cover the possible concentrations in plasma and feces samples with proper precision and accuracy. The limit of quantification (LOQ) was 0.625 μg/mL for butyric acid, and the lower limit of detection (LOD) was 0.3 μg/mL.

Standard butyric acid solutions were added to plasma and feces samples, and the increased peak area for butyric acid in the sample was compared with the standard curve to calculate the recovery. The recovery of butyric acid in feces was 99.15, 97.32, and 99.58%, and the results in plasma were 102.8, 101.52, and 99.62%. The accuracy, precision, and recoveries of the butyric acid results are shown in Table 1.

**Table 1:** Validation results of butyrate in plasma and feces by GC.

	**Plasma**			**Feces**		
	**LQC**	**MQC**	**HQC**	**LQC**	**MQC**	**HQC**
**ACCURACY AND PRECISION (*n* = 5)**						
Day1- Mean (μg/mL)	1.22	51.41	385.12	1.22	51.41	385.12
Day1- RE (%)	−2.40	2.82	−3.72	−2.40	2.82	−3.72
Day1- RSD (%)	5.42	1.40	0.17	5.42	1.40	0.17
Day2- Mean (μg/mL)	1.34	46.04	390.74	1.34	46.04	390.74
Day2- RE (%)	7.20	−7.92	−2.32	7.20	−7.92	−2.32
Day2- RSD (%)	4.14	3.16	2.00	4.14	3.16	2.00
Day3- Mean (μg/mL)	1.28	51.65	399.10	1.28	51.65	399.10
Day3- RE (%)	2.40	3.30	−0.22	2.40	3.30	−0.22
Day3- RSD (%)	7.48	1.59	0.73	7.48	1.59	0.73
**RECOVERY (*n* = 3)**						
Mean (%)	102.8	101.52	99.62	99.15	97.32	99.58
RSD (%)	4.93	0.07	0.27	6.64	3.05	4.22
**STABILITY AFTER PREPARATION (*n* = 3)**						
**At ambient temperature for 24 h**		**Un-spiked**	**Spiked**		**Un-spiked**	**Spiked**
Mean (%)	–	102.52	101.9	–	96.18	96.34
RSD (%)	–	1.56	1.23	–	3.05	1.11
**At –20°C for 48 h**		**Un-spiked**	**Spiked**		**Un-spiked**	**Spiked**
Mean (%)	–	98.48	100.57	–	99.81	99.1
RSD (%)	–	2.9	1.08	–	0.71	0.21

The stability results showed that both plasma and feces samples were stable, with values range from 96.2 to 102.5%. The long-term stability results showed that samples were stable under this condition, with values range from 98.5 to 100.6% in plasma and feces.

Therefore, a reliable, reproducible method for detecting butyrate in feces and plasma was developed and identified (Table 1).

#### Butyrate in beagle dog feces and plasma

The results are shown in Figure 3D. Treating beagle dogs with BBR (50 mg/kg) caused increased production of butyrate in a time-dependent manner. After continuous administration for 5 consecutive days, a statistically significant increase in both plasma (Figures 3D–L) and feces (Figures 3D–R) was observed (^*^p < 0.05). After 7 days of administration, butyrate in feces and plasma increased by 1.3- and 1.2-fold, respectively, compared with the levels in pre-treatment samples, with *p*-values of less than 0.01 and 0.001, respectively. This result suggests that BBR may induce the production of butyrate by regulating the abundance or function of gut microbiota in beagle dogs.

### Qualitative and quantitative determinations of BBR metabolites in plasma and feces by LC-MS/MS and LC/MS^n^-IT-TOF

#### Identification of BBR metabolites in dog plasma and feces

**Figure 4:**
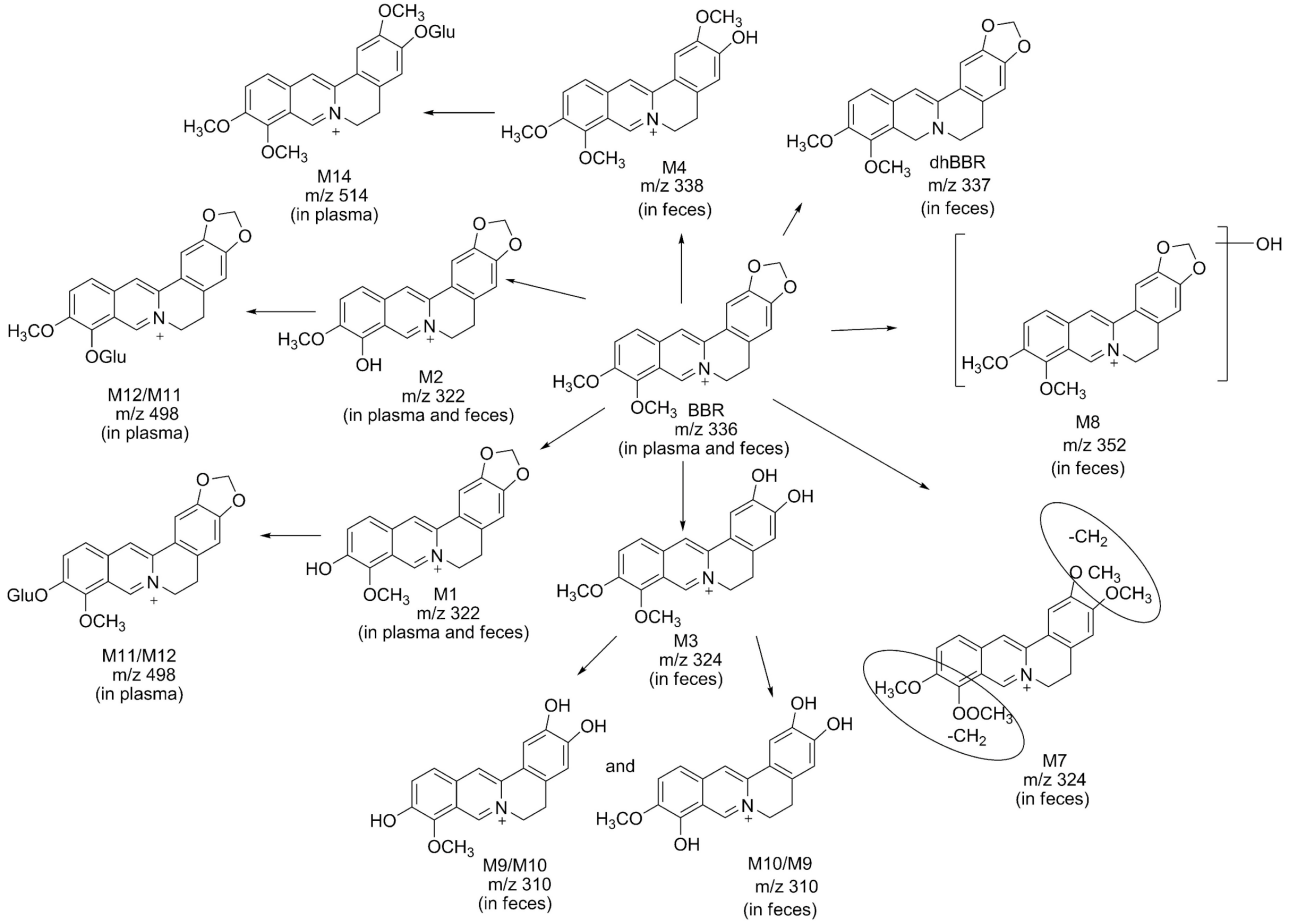
Possible metabolic pathways of BBR after administration of BBR to beagle dogs.

Eleven metabolites were identified in the present work. Structure of M1 (*m/z* 322), M2 (*m/z* 322), M3 (*m/z* 324), M4 (*m/z* 338), M5 (*m/z* 352), and M6 (*m/z* 338) were elucidated according to the MS/MS transitions and fragment pathways, also confirmed by the reference substances. Table 2 showed metabolites thalifendine (M1), berberrubine (M2), glucuronic acid conjugated thalifendine (M11), glucuronic acid conjugated berberrubine (M12) and glucuronic acid conjugated jatrorrhizine (M14), which were found in plasma (Figure 2E), and M1, M2, demethyleneberberine (M3), jatrorrhizine (M4), 3, 9-demethyl-palmatine (M7), hydroxylated berberine (M8), hydroxylated demethyleneberberine (M9 and M10), which were found in feces (Figure 2F). Eleven metabolites were detected in plasma and feces after BBR oral administration to dogs, including eight phase I metabolites (M1, M2, M3, M4, M7, M8, M9, and M10) and three phase II metabolites (M11, M12, and M14; Figure 4).

**Table 2:** Formula, retention time and MS/MS transitions data from the metabolites of berberine by LC-MS/MS and LC/MS^n^-IT-TOF in beagle plasma and feces.

**Compound**	**t_R_ (min)**	**Formula (M)**	**MS/MS transitions (m/z)**	**MS^1^[M]^+^**	**Fragments**				**Presented in plasma or/and feces**
					**MS^2^ m/z**	**MS^3^ m/z**	**MS^4^ m/z**	**MS^5^ m/z**	
M1	3.67	C_19_H_16_NO_4_	322→ 307	322	307	279	264	236	Plasma and feces
M2	3.93	C_19_H_16_NO_4_	322→ 307	322	307, 294	279, 250	263, 220	205, 234	Plasma and feces
M3	3.33	C_19_H_18_NO_4_	324→ 308	324	280, 309	265, 235	236, 219		Feces
M4	3.74	C_20_H_20_NO_4_	338→ 322	338	294, 323	307, 294	279		Feces
M7	3.23	C_19_H_18_NO_4_	324→ 309	324	309, 294	294, 238	266, 238	238, 210	Feces
M8	3.84	C_20_H_18_NO_5_	352→ 337	352	337, 308	290	232, 262, 204	204	Feces
M9	2.62	C_18_H_16_NO_4_	310→ 295	310	295, 249	267	251, 238, 213	222, 205	Feces
M10	2.82	C_18_H_16_NO_4_	310→ 295	310	295	267	238, 222	223	Feces
M11	2.90	C_25_H_24_NO_10_	498→ 322	498	322	–	–	–	Plasma
M12	3.12	C_25_H_24_NO_10_	498→ 322	498	322	–	–	–	Plasma
M14	2.88	C_26_H_28_NO_10_	514→ 338	514	338	–	–	–	Plasma

–*: Content of metabolites (M11, M12, and M14) in plasma is too low, and the sensitivity of LC/MS^n^-IT-TOF was not enough to detect them. So, the LC-MS/MS was used to identify the structures of metabolites in plasma*.

Metabolite M1 and M2 eluted at 3.67 and 3.93 min, respectively, with the same [M]^+^ *m*/*z* 322, 14 Da less than BBR. M1 and M2 were isomers, it could be the demethylated metabolites of BBR. Their retention time, MS/MS transitions and the fragment pathways were the same as that of reference substances. The fragments of *m/z* 307 (loss of CH_3_) on MS^2^, *m/z* 279 (loss of CH_3_ and CO) on MS^3^, *m/z* 263 (loss of 2CH_3_, CO, and H) on MS^4^, and *m/z* 234 (loss of 2CH_3_, 2CO, and 2H) on MS^5^ were observed. So, they were speculated as thalifendine (M1) and berberrubine (M2), respectively.

Metabolite M3 eluted at 3.33 min. [M] ^+^ m/z of M3 was 324, which was 12 Da less than that of BBR. The retention time, MS/MS transitions and the fragment pathways were consistent with standard substance of demethyleneberberine. The characteristic product ions were *m/z* 309 (loss of CH_3_) and *m/z* 280 (loss of CH_4_ and CO) on MS^2^ and MS^3^, respectively. The fragment of *m/z* 265 (loss of 2CH_3_, CO, and H) on MS^4^ was detected, and it could further fragment to *m/z* 236 (loss of 2CH_3_, 2CO, and 2H) on MS^5^.Therefore, M3 was identified as demethyleneberberine.

Metabolite M4 eluted at 3.74 min, with [M]^+^ of 338. The fragment signal at *m/z* 323 corresponded to loss of CH_3_ on MS^2^, and *m/z* 294 was formed by loss of CH_4_ and CO on MS^3^. The fragment on MS^4^ was 279 (loss of 2CH_3_, CO, and H). The retention time, MS/MS transitions and the fragment pathways were consistent with standard substance of jatrorrhizine. M4 was deduced as jatrorrhizine.

Metabolite M7, with a retention time of 3.23 min, exhibited [M]^+^ m/z 324, 12 Da less than BBR. M7 was an isomer of M3, and with a different fragmentation pathway from M3. The cleavage of *m/z* 309 was formed by loss of one CH_3_. The fragment of *m/z* 294 (loss of 2CH_3_) was examined on MS^3^. The fragment of *m/z* 266 (loss of 2CH_3_ and CO) and *m/z* 238 (loss of 2CH_3_ and 2CO) were observed on MS^4^ and MS^5^. M7 could be identified as 3,9-demethyl-palmatine.

Metabolite M8 eluted at 3.84 min, and showed a [M]^+^ at 352, 16 Da higher than BBR. MS^2^ presented *m/z* 337 (loss of CH_3_) and *m/z* 308 (loss of CH_3_ and COH). Fragment ion *m/z* 337 could further split to ions at 290 (loss of CH_3_, COH, and H_2_O) on MS^3^, *m/z* 262 (loss of CH_3_, 2CO, H, and H_2_O) on MS^4^, and *m/z* 204 on MS^5^. It could be hydroxylated occurred on BBR. M8 was presumed hydroxylated berberine.

Metabolites M9 and M10 were isomers, with retention times of 2.62 and 2.82 min, respectively, with a [M]^+^ at 310, 14 Da less than BBR. The fragments of *m/z* 295 (loss of CH_3_) on MS^2^, *m/z* 267 (loss of CH_3_ and CO) on MS^3^, and *m/z* 251 (loss of C_2_H_6_ and CO) on MS^4^ were detected. The structures of M9 and M10 were conjectured to be hydroxylated demethyleneberberine.

Metabolite M11 and M12 eluted at 2.90 and 3.12 min, respectively, with a [M]^+^ of 498, 176 Da higher than M1 and M2, indicating a glucuronidated M1 and M2. The neutral loss of 176 Da from parent ion gave the major fragment ion at *m/z* 322 on MS^2^. M11 and M12 were identified as the glucuronic acid conjugated thalifendine and glucuronic acid conjugated berberrubine.

Metabolite M14, with a retention time of 2.88 min showed a [M]^+^ *m/z* 514, 176 Da higher than M4. Therefore, M14 was identified as glucuronic acid conjugated jatrorrhizine.

#### Quantitation of BBR and its metabolites in plasma and feces

##### Validation of BBR and its metabolites in plasma by LC-MS/MS

**Figure 5:**
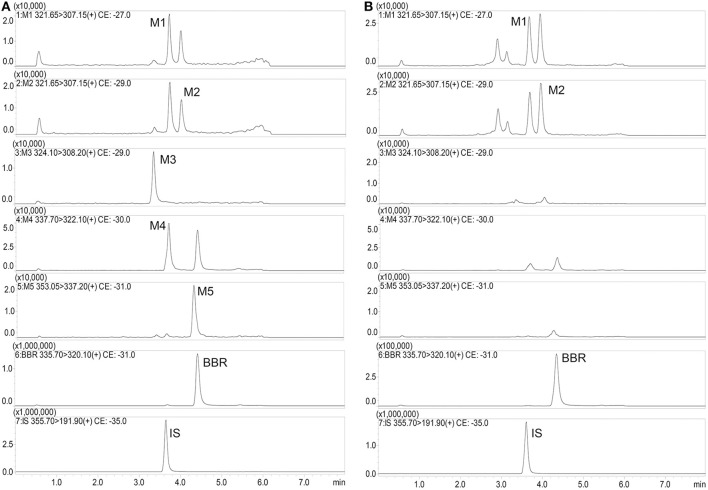
Typical mass spectra of BBR and its metabolites in beagle dog plasma (**A**: Spiked sample at the lower limits of quantitation; **B**: Plasma sample at 480 min after administration).

Typical mass spectra of BBR and its metabolites in beagle dog plasma are shown in Figure 5. The method was linear, with a weighing factor (1/c) in the range of 0.1–200 ng/mL for M1, M2, M3, M4, and M5, except for BBR, which was 0.2–200 ng/mL, with intra- and inter-day accuracy and precision within the acceptance criteria as per the FDA and EMA guidelines (Table 3). The mean regression coefficient was more than 0.99.

**Table 3:** Validation results of BBR and its metabolites in plasma by LC-MS/MS (*n* = 5).

	**M1**			**M2**			**M3**			**M4**			**M5**			**BBR**		
	**LQC**	**MQC**	**HQC**	**LQC**	**MQC**	**HQC**	**LQC**	**MQC**	**HQC**	**LQC**	**MQC**	**HQC**	**LQC**	**MQC**	**HQC**	**LQC**	**MQC**	**HQC**
**PRECISION AND ACCURACY**																		
**Batch 1**																		
Mean (ng/mL)	0.23	8.85	143.45	0.21	9.23	149.32	0.21	8.71	161.11	0.22	9.26	150.92	0.25	10.30	149.98	0.41	10.83	148.99
RE (%)	16.50	−11.53	−10.34	3.50	−7.68	−6.68	6.20	−12.95	0.69	8.20	−7.37	−5.67	23.75	2.97	−6.26	2.70	8.32	−6.88
RSD (%)	5.44	6.00	5.43	5.66	11.04	6.31	16.18	4.63	8.27	13.59	7.08	10.74	18.34	7.44	10.16	13.22	9.19	12.16
**Batch 2**																		
Mean (ng/mL)	0.24	9.69	175.31	0.25	10.75	183.81	0.23	9.40	176.65	0.24	10.25	155.81	0.23	11.33	161.03	0.47	10.15	147.24
RE (%)	18.20	−3.12	9.57	25.60	7.54	14.88	12.50	−6.00	10.41	19.30	2.53	−2.62	16.90	13.28	0.64	16.90	1.47	−7.97
RSD (%)	12.06	8.67	9.90	15.59	7.66	4.55	14.46	13.05	4.68	15.18	3.83	7.46	11.89	3.62	13.57	17.28	12.30	10.48
**Batch 3**																		
Mean (ng/mL)	0.23	9.33	168.04	0.23	8.87	177.25	0.23	8.61	165.84	0.24	9.93	165.70	0.24	11.06	175.62	0.40	10.71	166.39
RE (%)	14.42	−6.66	5.03	14.00	−11.32	10.78	12.58	−13.88	3.65	20.00	−0.66	3.56	18.42	10.59	9.76	1.00	7.14	3.99
RSD (%)	10.04	10.03	10.55	15.76	9.53	10.48	17.08	6.40	6.72	19.33	6.01	7.69	8.51	2.51	9.17	14.10	8.27	12.16
**RECOVERY (%)**																		
Mean	117.68	100.27	96.19	117.21	113.34	96.38	115.33	106.30	96.30	103.74	99.36	95.54	97.40	101.79	97.13	104.41	117.48	111.82
RSD (%)	5.98	1.57	2.70	18.66	3.42	4.25	14.77	6.30	5.59	5.18	2.86	3.06	6.53	6.00	5.49	13.41	8.88	11.13
**MATRIX EFFECT (%)**																		
Mean	93.04	101.14	103.57	101.49	89.61	104.93	90.72	96.69	108.73	99.19	100.32	104.98	95.54	103.16	102.91	94.65	97.06	101.46
RSD (%)	5.59	2.83	4.99	13.25	3.57	5.70	6.56	6.77	7.27	3.60	2.40	3.90	4.84	3.88	5.03	14.69	7.22	5.50
**STABILITY OF PLASMA BEFORE PREPARATION**																		
**At ambient temperature for 24 h**																		
Mean (ng/mL)	0.19	9.25	179.45	0.18	9.00	164.96	0.22	9.10	171.01	0.22	10.54	173.35	0.20	11.05	178.42	0.42	10.15	172.62
RE (%)	−3.33	−7.48	12.16	−9.70	−10.02	3.10	10.50	−8.97	6.88	8.20	5.43	8.35	−1.10	10.53	11.51	5.15	1.49	7.89
RSD (%)	14.07	9.61	9.65	6.25	6.47	12.11	12.19	5.38	6.90	12.14	8.41	11.54	12.12	2.28	10.56	16.88	11.07	13.10
**At −20°C for 72 h**																		
Mean (ng/mL)	0.21	10.02	161.49	0.19	9.71	175.16	0.20	10.03	161.87	0.21	11.20	176.94	0.23	11.30	173.93	0.40	9.45	180.78
RE (%)	6.83	0.24	0.93	−5.75	−2.90	9.47	1.67	0.32	1.17	2.83	11.99	10.59	13.08	12.97	8.70	1.17	−5.50	12.99
RSD (%)	14.61	9.14	9.54	6.93	13.04	7.49	15.79	3.18	14.63	10.44	6.57	8.45	13.27	10.00	11.46	5.84	11.53	6.26
**After three freeze–and–thaw cycles**																		
Mean (ng/mL)	0.20	9.61	153.88	0.20	9.30	152.79	0.21	8.69	162.96	0.20	10.10	158.08	0.21	10.58	159.00	0.38	9.62	153.31
RE (%)	0.40	−3.85	−3.83	0.50	−7.01	−4.51	3.20	−13.14	1.85	1.60	0.96	−1.20	4.10	5.78	−0.63	−5.65	−3.84	−4.18
RSD (%)	7.31	4.69	6.42	8.82	10.22	14.36	8.63	6.27	6.79	8.77	5.53	8.30	3.92	7.59	8.08	5.34	11.62	12.69
**STABILITY OF PLASMA AFTER TREATMENT IN THE AUTOSAMPLER FOR 24 h AT 4°C**																		
Mean (ng/mL)	0.23	9.21	176.69	0.22	8.73	179.77	0.22	8.75	160.78	0.20	10.59	166.52	0.22	0.23	9.21	176.69	0.22	8.73
RE (%)	16.50	−7.86	10.43	12.33	−12.68	12.36	7.75	−12.53	0.48	−0.25	5.93	4.08	8.00	16.50	−7.86	10.43	12.33	−12.68
RSD (%)	13.05	7.37	9.63	6.27	7.91	11.65	13.35	3.28	5.78	8.43	6.40	10.42	11.57	13.05	7.37	9.63	6.27	7.91

The lower limit of quantification was 0.1 ng/mL for M1 (RE: 17.67%, RSD: 13.01%), M2 (RE: 19.40%, RSD: 1.52%), M3 (RE: 0.83%, RSD: 12.57%), M4 (RE: −7.60%, RSD: 8.54%) and M5 (RE: 14.50%, RSD: 19.08%), and the LLOQ of BBR (RE: 11.50%, RSD: 14.73%) was 0.2 ng/mL.

The percentage recovery (RE) and matrix effect (ME) were in the range of 94.38–117.68% and 89.61–108.73%, respectively, with RSD meeting the bioanalysis requirements.

Table 3 shows the stability results before preparation, including plasma at ambient temperature for 24 h, −20°C for 72 h, and after three freeze-and-thaw cycles, as well as plasma samples prepared in the autosampler for 24 h at 4°C. All of the assay validation parameters were within the acceptable limits.

Thus, the precision, accuracy, recovery, matrix effect and stability tests met the criteria for quantitative determination in biological samples.

##### Quantitation of BBR and its metabolites in plasma and feces

The pharmacokinetic profile of BBR is represented in Figures 3A–L and Table 4. According to the data as analyzed by DAS 3.0, the value of *t*_1/2_ was 552.15 ± 269.50 min. The value of AUC _(0−48h)_ was 13175.62 ± 5426.29 ng/mL^*^min. There could be the enterohepatic circulation after oral administration with BBR to beagle dogs since that multiple peaks could be observed from the plasma concentration-time curve (Figures 3A–L). The value of *C*_*max*_was 36.88 ± 23.45 ng/mL in this study.

**Table 4:** Pharmacokinetic parameters of BBR after oral administration to male beagle dogs (50 mg/kg, Mean ± SD, *n* = 5).

**Parameters units**	**Mean ± SD**
*AUC_(0−48*h*)_* ng/mL^*^min	13175.62 ± 5426.29
*AUC*_(0−∞)_ *n*g/mL ^*^min	13850.39 ± 6180.73
*MRT*_(0−48*h*)_ min	651.37 ± 159.51
*MRT*_(0−∞)_ min	771.83 ± 295.15
*t*_1/2*z*_ min	552.15 ± 269.50
*C_*max*_* ng/mL	36.88 ± 23.45

The relative contents of M11 were the highest compared with M1, M2, M12, and M14, with a *C*_*max*_ of 5.63 ± 3.07 ng/mL at 240 min in plasma (Figures 3A–R). The *C*_*max*_ of metabolites was as follows: M11 > M2 [(0.89 ± 0.56) ng/mL] > M14 [(0.82 ± 0.21) ng/mL] > M1 [(0.64 ± 0.59) ng/mL] > M12 [(0.44 ± 0.20) ng/mL] (Figures 3A–R).

After oral administration of BBR to male beagle dogs, the highest amount of BBR was detected in feces. The contents were 2625.04 ± 1726.94 μg/g and 2793.43 ± 488.10 μg/g on days 3 and 7 (Figures 3C–L), respectively. Furthermore, the plasma concentration (Figure 3B) and excretion amounts (Figures 3C–L,C–M) of BBR and its metabolites on days 3 and 7 were equivalent.

### Bacterial composition analysis

**Figure 6:**
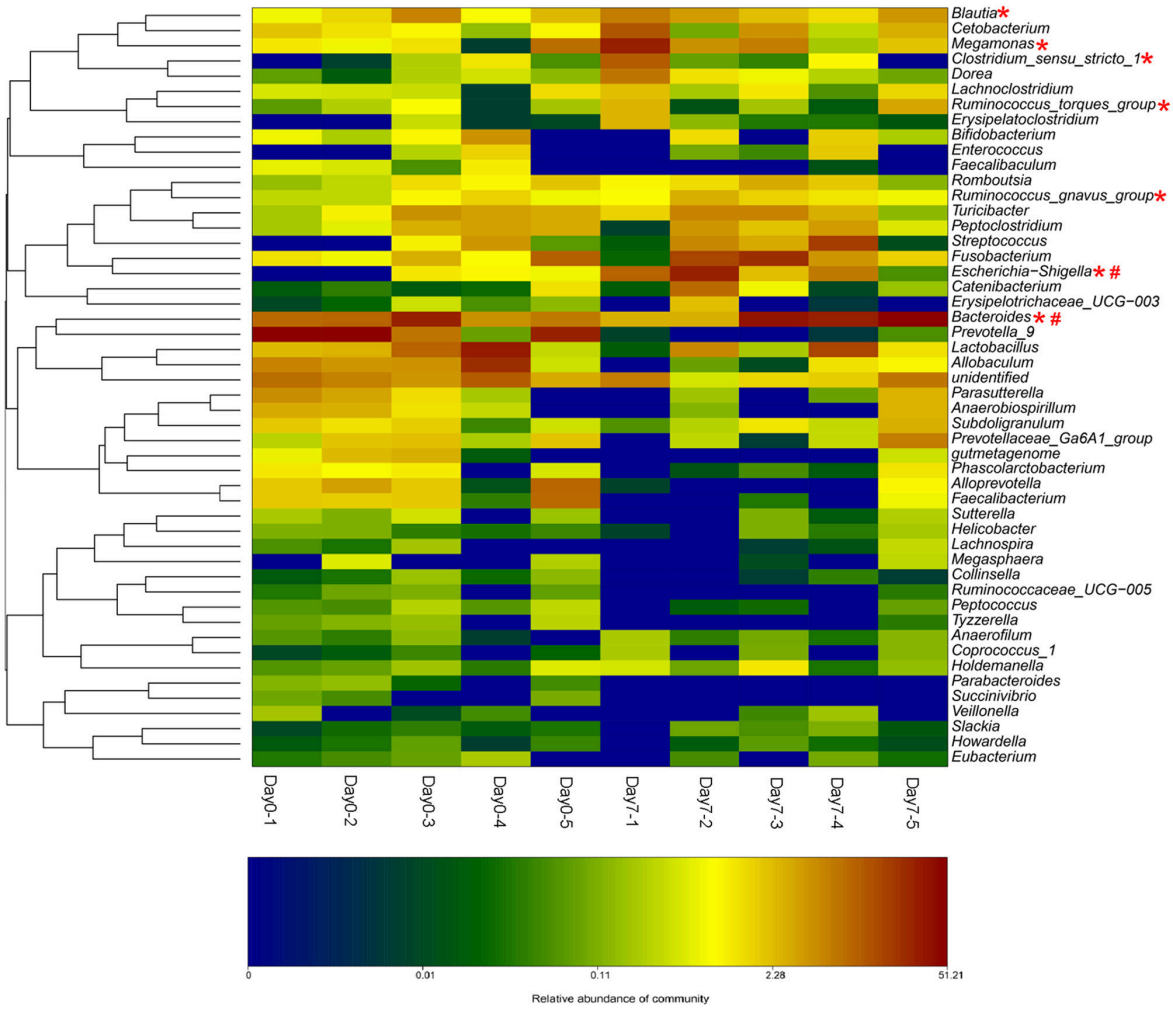
BBR modified the intestinal bacteria composition by increasing the abundance of beagle dogs (The heat-map shows the top 50 bacterial genera with the most substantial change in abundance after exposure to BBR. The color of the spot corresponds to the normalized and log-transformed relative abundance of genera. The change of color from green to red represents corresponding colony abundance. ^*^Butyrate-producing bacteria; # Nitroreductases-producing bacteria).

The beagle dogs were treated orally with BBR (50 mg/kg/day) for 7 days and their feces samples were collected for the bacterial composition analysis. The barcoded pyrosequencing of the V3 and V4 regions of the 16S rRNA gene showed that BBR enriched the abundance of butyrate-producing bacteria in the dog intestine. The heatmap of the top 50 bacterial genera that exhibited the most substantial change in abundance after exposure to BBR was shown in Figure 6. Of the 50 genera, the abundance of seven genera which were reported to be related with the production of butyrate increased, suggesting that the increased production of butyrate was result from the increased abundance of the related bacteria.

## Discussion

The central role of the intestinal microbiota in the progression and, more and more attention has been paid to the relationships between intestinal microbiota and host. As we all know that the gut microbiota could produce an extremely diverse metabolite repertoire from the anaerobic fermentation of exogenous undigested dietary components, as well as endogenous compounds that are generated by microorganisms and the host (Rooks and Garrett, 2016). In order to demonstrate the interactions between intestinal microbiota and xenobiotics, we tried to describe gut microbiota-regulated pharmacokinetics in beagle dogs after oral administration of BBR by single or multiple doses for 7 days in the present work.

### Biotransformations of BBR by intestinal microbiota

The enteric microbiota within the gastrointestinal lumen has the metabolic activity equal to a virtual organ. Gene diversity among the microbial community provides a variety of enzymes and biochemical pathways that are distinct from the host's own constitutive resources. DhBBR is a transient form of BBR in the intestinal lumen with improved physiochemical characteristics for absorption. The gut microbiota can reduce BBR into its absorbable form, dhBBR, which then oxidizes back to BBR after absorption into intestinal tissues and enters the blood, as shown in our previous study (Feng et al., 2015). Thus, the present results from beagle dogs also displayed that dhBBR is a gut-bacteria metabolite that can be synthesized from BBR in the intestinal environment.

### Effects of BBR on intestinal microbiota

Recent studies have shown that BBR might modulate the composition of the intestinal bacterial community (Zhang et al., 2012). The work found that the prevention of obesity and insulin resistance by berberine in HFD-fed rats is at least partially mediated by structural modulation of the gut microbiota. Short chain fatty acid (SCFA), is the principle product of the bacterial fermentation of non-digestible carbohydrates in gut, which could then be absorbed to blood, has been reported to regulate host energy metabolism and benefit health (Byrne et al., 2015). Among which, butyrate has been focused most extensively (Leonel and Alvarezleite, 2012; Koh et al., 2016), and a-butyrate-enriched high fat diet has shown an increased thermogenesis and energy expenditure. Supplementation of butyrate on the high-fat diet prevented development of insulin resistance and obesity in C57BL/6 mice (Gao et al., 2009). Our previous studies have demonstrated that oral administration of BBR in animals might promote gut microbiota production of butyrate through the acetyl CoA-butyryl pathway, which then enters the blood and reduces blood lipid and glucose levels (Wang et al., 2017a). Therefore, butyrate, a bioactive metabolite of the gut microbiota, was first selected as a biomarker to study the pharmacokinetic behavior of BBR, and butyrate showed high levels *in vivo* after oral treatment of beagle dog with BBR, a result that is related to the anti-hyperlipidemia effect of BBR.

From the bacterial composition analysis, we found that the abundance of seven butyrate-producing genera increased after 7 days BBR treatment in beagle dogs. *Escherichia-Shigella* (Varrone et al., 2015; Wang et al., 2017a), *Clostridium sensu stricto 1* (Colin et al., 2001; Wang et al., 2017a), *Megamonas* (Sandri et al., 2016), *Bacteroides* (Takahashi et al., 2016; Wang et al., 2017a), *Ruminococcus* (Takahashi et al., 2016) (gnavus and torques groups) and *Blautia* (Sandri et al., 2016) could produce butyate. Among which, *Escherichia-Shigella* has the biggest increase. The increased abundance of the butyrate-producing bacteria by BBR represents a favorable action of the drug on these bacteria. From our previous study (Wang et al., 2017a), the increased abundance of *Escherichia-Shigella, Clostridium sensu stricto 1*, and *Bacteroides* might be a reason for the elevation in butyrate production. Results of fecal microbiome in healthy dogs reported by Sandri (Sandri et al., 2016) indicated that positive correlations with butyrate production could be obtained for the *Blautia* and *Megamonas*.

Moreover, the increased abundance of nitroreductases producing bacteria was observed in *Escherichia–Shigella* (Fu et al., 2007) and Bacteroides (Schapiro et al., 2004). They were also found as the nitroreductases-producers in the intestinal bacterial community from our preliminary investigations (Wang et al., 2017b). Nitroreductases in bacterial could reduce BBR into dhBBR as an intestine-absorbable form (Feng et al., 2015).

These results suggested that treating dogs with BBR for 7 days could increase both the abundance of the butyrate- and the nitroreductases- producing bacteria.

### Pharmacokinetics of BBR in beagle dogs

Eleven metabolites firstly found in beagle dogs are in accordance with the findings of our previous studies (Ma et al., 2013; Tan et al., 2013), in which 16 metabolites were identified in rat bile, urine and feces after BBR administration by LC/MS^n^-IT-TOF. M5, M6, M13, M15, and M16 were not detected in beagle dogs. The reason could be that there are species differences between animals with regards to isoform composition, expression and catalytic activities of drug-metabolizing enzymes (Martignoni et al., 2006).

Phase I metabolites were widely distributed in plasma and feces. Phase II metabolites were glucuronic acid conjugated thalifendine (M11/M12) and glucuronic acid conjugated jatrorrhizine (M14), which were found in plasma. BBR is not primarily excreted in the form of Phase II metabolites in feces after administration. It is suggested that BBR and its Phase I metabolites are isoquinoline alkaloids, and they could be excreted more easily than other natural products because of their electron-deficient state. Therefore, conjugated metabolites does not account for the primary excretion of BBR (Ma et al., 2013).

Preliminary pharmacokinetic studies indicated that BBR has poor oral bioavailability of less than 1% (Shen et al., 1993; Yu et al., 2000; Zuo et al., 2006; Liu et al., 2010). In this study, we also found that the concentration of BBR in plasma was very low after BBR administration to beagle dogs.

BBR was mainly metabolized *in vivo* to generate M1 and M1 glucuronide via oxidative demethylation and subsequent glucuronidation and then to form M2 and M2 glucuronide via oxidative demethylation and subsequent glucuronidation, which was consistent with previous studies (Tsai and Tsai, 2004; Qiu et al., 2008; Liu et al., 2009). Our results also demonstrated that UDP-glucuronosyltransferase was the major drug-metabolizing enzyme responsible for the formation of phase II metabolites of berberine. The peak time of phase II metabolites was 240 min, while that of phase I metabolites was 360 min. The reason for this difference could be that the biotransformation and conjunction reactions occurred at the same time.

Therefore, extensive elimination may be another cause of PK, resulting in low plasma concentrations of BBR in dogs. Although BBR itself is the most active form for anti-hyperlipidemia, its metabolites remain active with 30–70% activity (Li et al., 2011). For instance, berberrubine and thalifendine are the two active metabolites, which could up-regulate LDLR and adenosine 5'-monophosphate-activated protein kinase (AMPK) activation, but with reduced potency (Li et al., 2011). So, the potential bioactivities of the abundant metabolites *in vivo* might also be a mechanism of BBR action of anti-hyperlipidemia effect. These results might explain the discrepancy between the significant pharmacological effects and low bioavailability of BBR.

## Conclusions

In summary, we firstly explored the interactions between intestinal microbiota and xenobiotics in beagle dogs after oral administration of BBR by GC-MS, GC, LC-MS/MS, LC/MS^n^-IT-TOF, 16S rRNA genes analysis and QIIME methods. Extensive elimination may result in low plasma concentrations of BBR in dogs in this study. BBR could not only be converted into dhBBR by intestinal microbiota but also could promote the gut microbiota to produce butyrate, which is related to the anti-hyperlipidemia effect of BBR. The results might offer a partial explanation for the seemingly contradictory results between the desired effects and low bioavailability of BBR, which could be helpful for mechanistic and clinical studies.

## Ethics statement

This study was carried out in accordance with the recommendations of guidelines for Animal Experimental Center, Animal Care and Welfare Committee, Institute of Materia Medica, CAMS and PUMC. The protocol was approved by the Animal Care and Welfare Committee, Institute of Materia Medica, CAMS PUMC.

## Author contributions

Participated in the research design: YW, J-DJ, RF, and S-RM. Conducted the experiments: RF, S-RM, Z-XZ and FG. Performed the data analysis: YW, RF and S-RM. Wrote or contributed to the writing of the manuscript: YW, RF, Z-XZ, and S-RM.

### Conflict of interest statement

The authors declare that the research was conducted in the absence of any commercial or financial relationships that could be construed as a potential conflict of interest.

## Abbreviations

BBR: berberine
dhBBR: dihydroberberine
GC: gas chromatography
GC-MS: gas chromatography-mass spectrometry
HQC: high concentration of quality control
IS: internal standard
LC/MS^n^-IT-TOF: High-performance liquid chromatography-ion trap-time of flight mass spectrometry
LC/MS-MS: liquid chromatography-triple quadrupole mass spectrometry
LDL-*c*: low-density-lipoprotein cholesterol
LOD: limits of detection
LOQ: limits of quantitation
LQC: low concentration of quality control
MQC: median concentration of quality control
PK: pharmacokinetics
SCFA: short-chain fatty acid
SD: standard deviation
TC: cholesterol
TG: triglyceride
OTU: operational taxonomic unit
QIIME: quantitative insights into microbial ecology.
